# Supplementary material for: Age, adrenal steroids, and cognitive functioning in captive chimpanzees (Pan troglodytes)
Source: PeerJ. 2022 Nov 9;10:e14323. doi: 10.7717/peerj.14323 (PMC9653054; doi:10.7717/peerj.14323)
Supplement: Supplemental Information 1 — We used the full dataset (N = 107) for comparison with the reduced dataset (main file) that controls for age discrepancy between hormonal and cognitive data. [file peerj-10-14323-s001.docx]

**Supplementary material**

For comparative purposes with the reduced dataset, we conducted Generalized Linear Model (GLM) analyses with the full dataset (N = 107) to compare the effect of age and adrenal steroids on chimpanzee cognition, as described in the methods.

The PCTB PC1 (*spatial relationships*) model with the lowest AICc included a significant interaction between age and the DHEAS/cortisol ratio (β ± SE = 0.002 ± 0.0007, t = 2.95, p = 0.004), which explained 10% of the variance in PC1. The presence of this interaction suggests that performance on *spatial relationships* tasks has a positive correlation with the DHEAS/cortisol ratio in older, but not younger individuals (Fig. S1). DHEAS and sex did not improve the model. The second set of PC1 models that included age, sex, cortisol, and their interactions as initial predictors were not better than the null model, indicating that cortisol alone was not associated with PC1.

FIGURE S1

The PCTB PC2 (*tool use and social communication*) model with the lowest AICc included a significant negative effect of age (β ± SE = -0.026 ± 0.0086, t = -3.02, p = 0.003), which explained 8% of the variance in PC 2 (Fig. S2). Although a second model not significant different than the best model included DHEAS as fixed factor (⍙AICc = 0.51), the effect was not significant (β ± SE = -0.0002 ± 0.0002, t = -1.27, p = 0.21), indicating that PC2 had no effect of DHEAS/cortisol ratio, DHEAS, or their interactions. Similarly, when we tested cortisol in a separate model that also included age, sex, as well as their interactions, we found that cortisol was not significantly correlated with PC2 (β ± SE = -0.002 ± 0.003, t = 0.78, p = 0.44) and therefore did not improve the model.

FIGURE S2

The PCTB PC3 (*auditory and visual sensory perception*) model with the lowest AICc included a non-significant, negative correlation with age (β ± SE = -0.02 ± 0.009, t = -1.7, p = 0.09) and it was not significantly different than the null model (⍙AICc = 0.9). However, when we tested cortisol separately from the other hormonal measures, the model with the lowest AICc included a significant negative effect of age (β ± SE = -0.02 ± 0.01, t = -2.04, p = 0.04), which explained for 2.8% of the PC3 variation (Fig. S3-A) and a significant negative effect of cortisol (β ± SE = -0.006 ± 0.003, t = -1.94, p = 0.05), which explained 3.5% of the PC3 variation (Fig. S3-B). A second model not different than the best model (⍙AICc = 1.66) included only age as predictor, but this effect was non-significant (β ± SE = -0.02 ± 0.01, t = -1.68, p = 0.09).

FIGURE S3
