# Supplementary figures and images for: Age, adrenal steroids, and cognitive functioning in captive chimpanzees (Pan troglodytes)
Source: PeerJ. 2022 Nov 9;10:e14323. doi: 10.7717/peerj.14323 (PMC9653054; doi:10.7717/peerj.14323)

**Spatial relationships (PC1)**

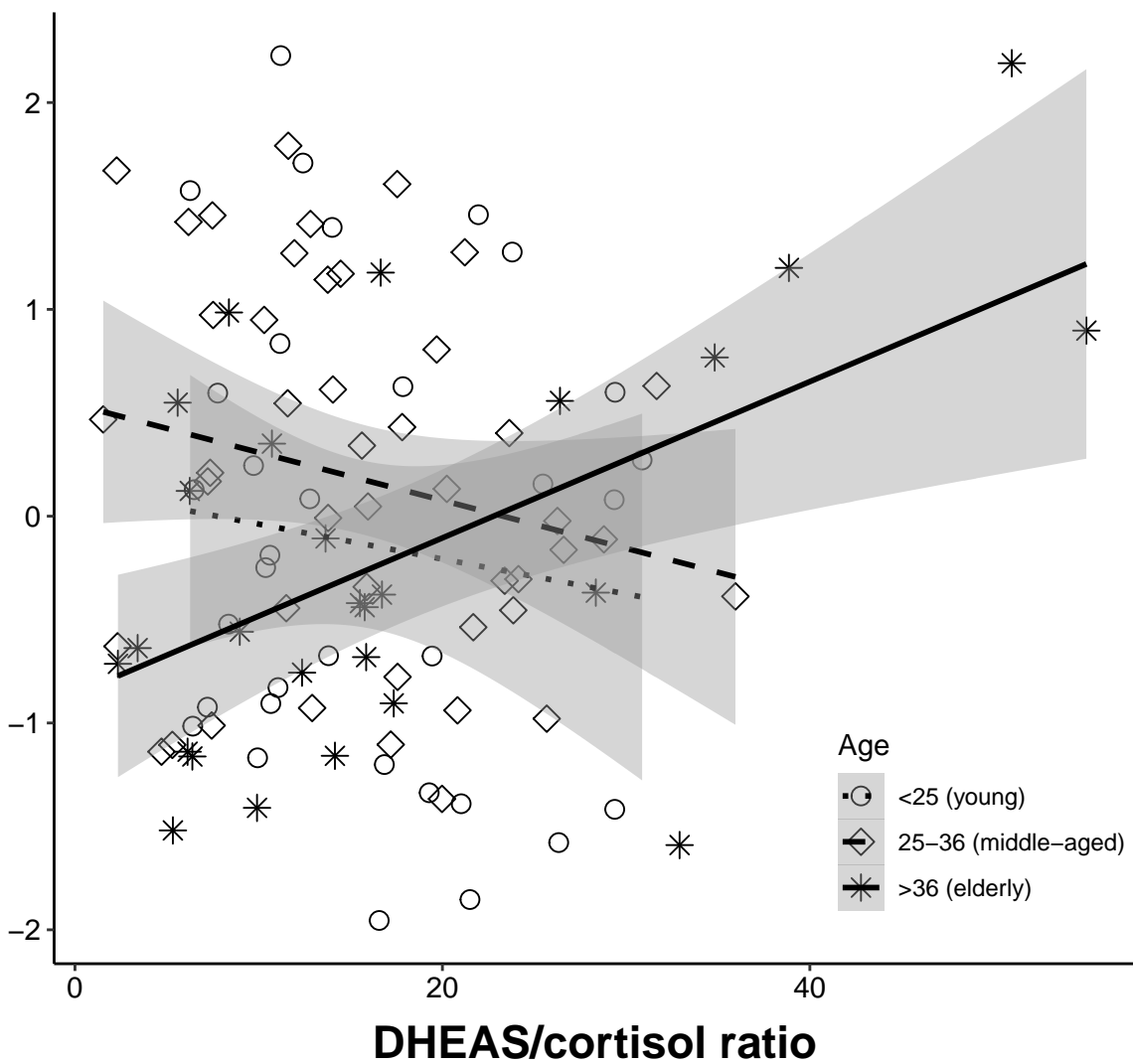

Supplement: Supplemental Information 2 — Each data point represents one individual (N = 107). The regression lines represent the predicted relationship between PC1 and DHEAS levels in three age categories: young (dotted line), middle-aged (dashed line), and elderly (solid line). The shaded areas represent a 95% confidence interval on the fitted values. [file peerj-10-14323-s002.pdf]

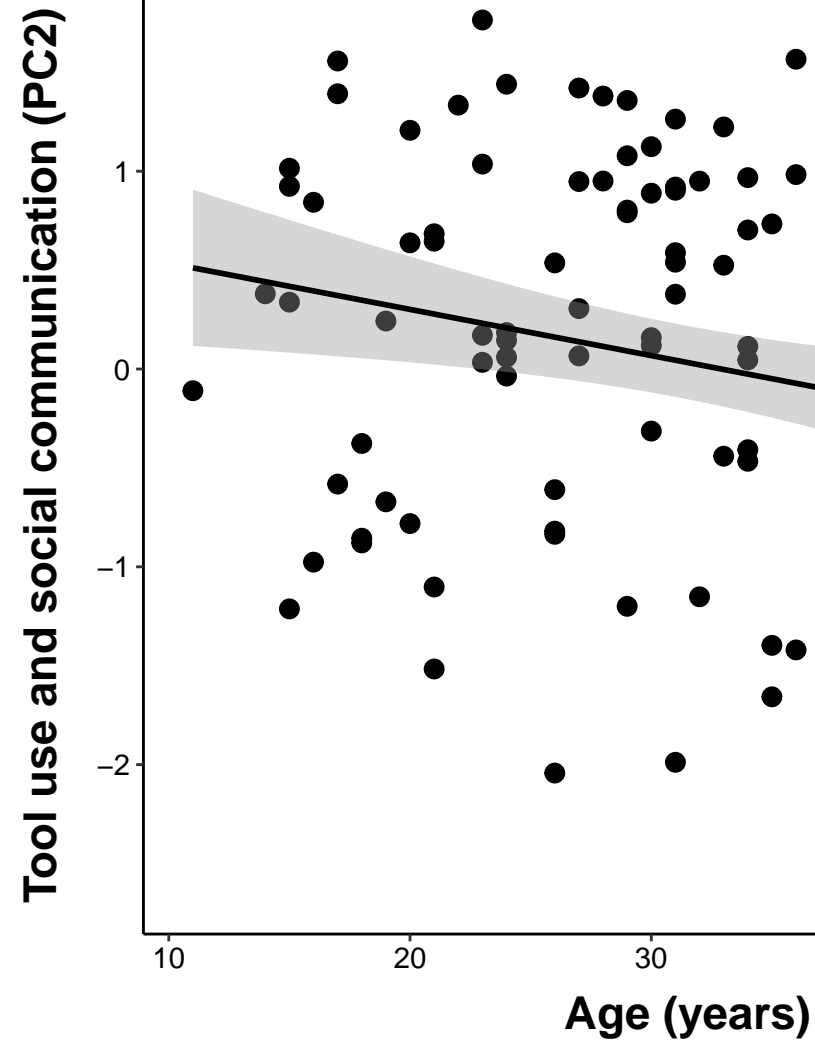

Supplement: Supplemental Information 3 — Each data point represents one individual (N = 107). The regression line represents the predicted relationship between PC2, and the shaded area represents a 95% confidence interval on the fitted values. [file peerj-10-14323-s003.pdf]

**Auditory and visual sensory perception (PC3)**

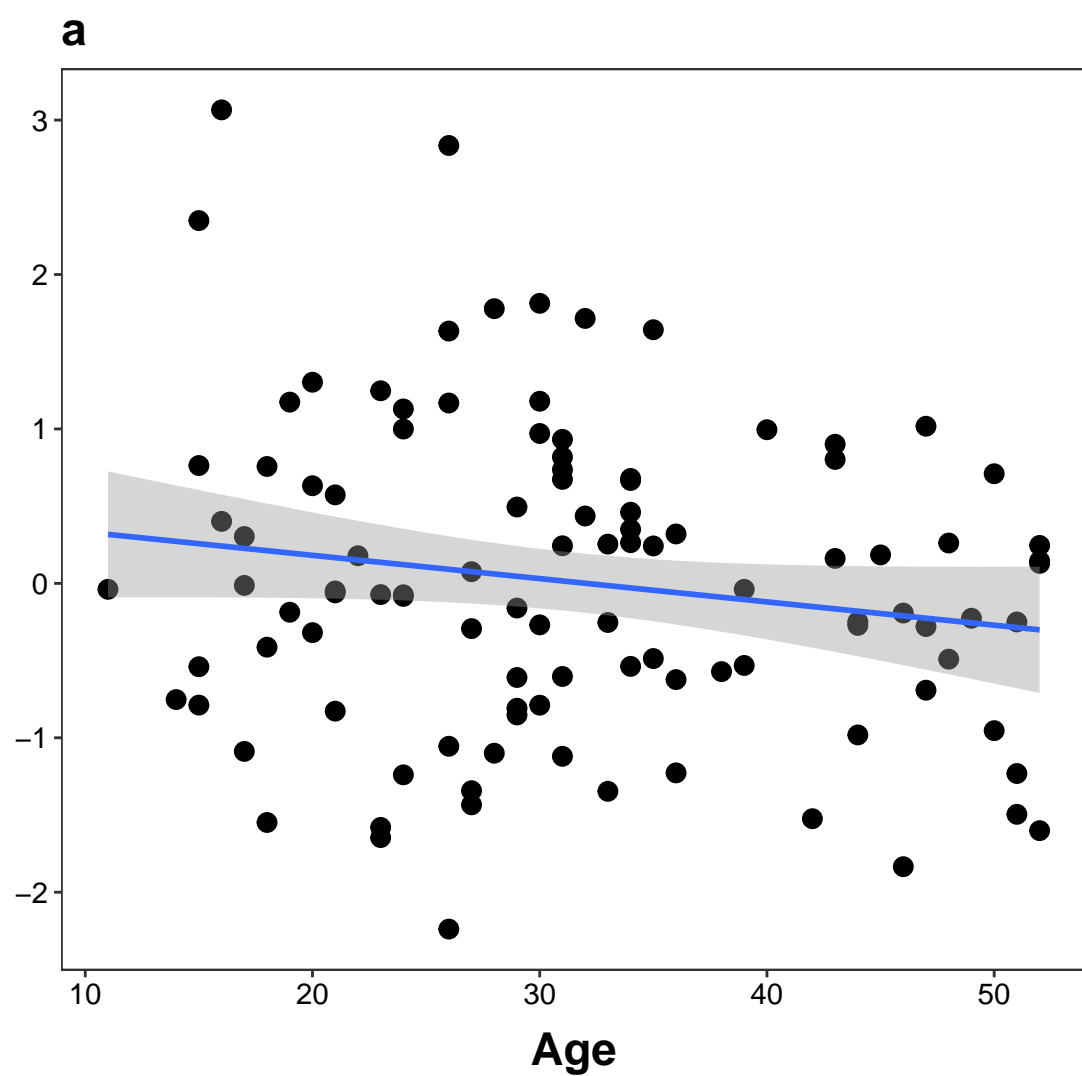

**Auditory and visual sensory perception (PC3)**

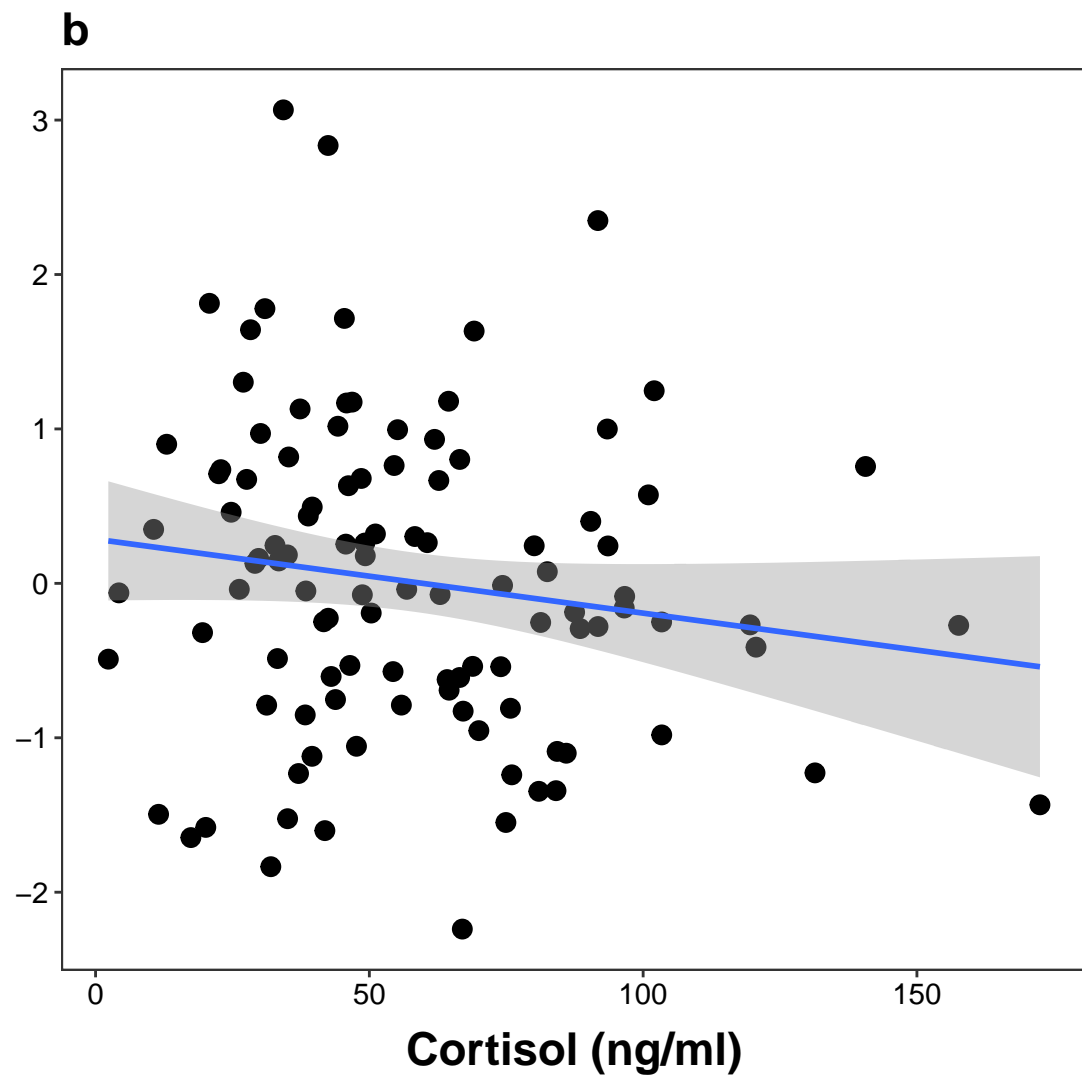

Supplement: Supplemental Information 4 — Each data point represents one individual (N = 107). The regression lines represent the predicted relationship between PC3 and (a) age or (b) cortisol levels. The shaded areas represent a 95% confidence interval on the fitted values. [file peerj-10-14323-s004.pdf]
